# Investigations by Cell-Mediated Immunologic Tests and Therapeutic Trials With Thymopentin in Vaginal Mycoses

**DOI:** 10.1155/S1064744996000439

**Published:** 1996

**Authors:** Werner Mendling, Ursula Koldovsky

**Affiliations:** 1 Department of Gynecology and Obstetrics Wuppental St. Antonius Hospital Düsseldorf Heinrich Heine Uiversität Düsseldorf Germany; 2 Department of Gynecology and Obstetrics Düsseldorf Heinrich Heine Uiversität Düsseldorf Germany; 3 Klinik für Frauenheilkunde und Geburtshilfe Klinikum Frankfurt (Oder) Müllroser Chaussee 7 Frankfurt (Oder) Markendorf D-15236 Germany

## Abstract

*Objective:* According to unsatisfactory therapeutic results in patients with chronically recurrent vaginal candidosis, we investigated if immunologic patient factors could be found and treated.

*Methods:* In 42 women with chronically recurrent and 20 women with acute *Candida albicans* vulvovaginitis, as well as 14 women with *C. glabrata* vaginitis, the following investigations were carried out: identification of yeast species; quantification of T lymphocytes and their subpopulations in sera; proliferation tests of T lymphocytes in vitro; treatment of 18 patients with chronically recurrent vaginal candidosis with the synthetic T-lymphocyte- stimulator thymopentin; and, finally, control of the above-mentioned parameters in the clinical course.

*Results:* Women with *C. albicans* vulvovaginitis showed fewer T lymphocytes and subpopulations in the peripheral blood than healthy women. Only the number of non-specific killer (NK) cells, however, was significantly lower in cases of acute *C. albicans* vulvovaginitis. In women with *C. glabrata* vaginitis, the number of T lymphocytes in the blood was within the normal range. In vitro proliferation tests using mitogens, bacterial antigens, and commercially available candida antigens with and without addition of thymopentin were carried out on the T lymphocytes of women with chronically recurrent *C. albicans* vulvovaginitis. These tests revealed no significant differences compared with the other patients with *C. albicans* infections. The patients were treated with thymopentin. Those women who revealed an increase of initially low numbers of T-helper cells recovered from vaginal candidosis after thymopentin treatment.

*Conclusions:* The peripheral T lymphocytes may be diminished in patients with chronically recurrent *C. albicans* vaginitis, and immunologic treatment can reduce the relapse rate.


Infectious Diseases in Obstetrics and Gynecology 4:225–231 (1996)
© 1997 Wiley-Liss, Inc.

### Investigations by Cell-Mediated Immunologic Tests and Therapeutic Trials With Thymopentin in Vaginal Mycoses

**Werner Mendling and Ursula Koldovsky**
*Department of Gynecology and Obstetrics, Wuppental St. Antonius Hospital (W.M.), and Department of Gynecology and Obstetrics (U.K.), Düsseldorf Heinrich Heine Universität, Düsseldorf, Germany*

### Abstract

*Objective:* According to unsatisfactory therapeutic results in patients with chronically recurrent vaginal candidosis, we investigated if immunologic patient factors could be found and treated.

*Methods:* In 42 women with chronically recurrent and 20 women with acute *Candida albicans* vulvovaginitis, as well as 14 women with *C. glabrata* vaginitis, the following investigations were carried out: identification of yeast species; quantification of T lymphocytes and their subpopulations in sera; proliferation tests of T lymphocytes in vitro; treatment of 18 patients with chronically recurrent vaginal candidosis with the synthetic T-lymphocyte- stimulator thymopentin; and, finally, control of the above-mentioned parameters in the clinical course.

*Results:* Women with *C. albicans* vulvovaginitis showed fewer T lymphocytes and subpopulations in the peripheral blood than healthy women. Only the number of non-specific killer (NK) cells, however, was significantly lower in cases of acute *C. albicans* vulvovaginitis. In women with *C. glabrata* vaginitis, the number of T lymphocytes in the blood was within the normal range. In vitro proliferation tests using mitogens, bacterial antigens, and commercially available candida antigens with and without addition of thymopentin were carried out on the T lymphocytes of women with chronically recurrent *C. albicans* vulvovaginitis. These tests revealed no significant differences compared with the other patients with *C. albicans* infections. The patients were treated with thymopentin. Those women who revealed an increase of initially low numbers of T-helper cells recovered from vaginal candidosis after thymopentin treatment.

*Conclusions:* The peripheral T lymphocytes may be diminished in patients with chronically recurrent *C. albicans* vaginitis, and immunologic treatment can reduce the relapse rate.
© 1997 Wiley-Liss, Inc.

Key words
*Candida albicans, Candida glabrata,* vulvovaginitis, cellular immunity, proliferation tests

Owing to particular pathogenic features, the facultatively pathogenic yeast *Candida albicans* is more likely to cause an infection in predisposed persons than other candida species are. These pathogenic features[1,2] include the utilization of carbohydrates and proteins, high-temperature and pH tolerance, occurrence in the guise of blastospores and pseudomycelia, formation of proteases and phospholipases, utilization of the host's ferrum by means of siderophores, capability of adherence to other epithelia, molecular mimicry to deceive immunocells, phenotype "switching," and possible synergism with bacteria. The clinical manifestations of vulvovaginal candidosis have been illustrated in a nomenclature recommendation.[3] Accordingly, *C. glabrata* vaginitis is usually associated with fewer clinical symptoms of vaginitis than *C. albicans* vulvovaginitis is.[4] Concerning the clinical signs, we do

Address correspondence/reprint requests to Dr. Werner Mendling, Klinik für Frauenheilkunde und Geburtshilfe, Klinikum Frankfurt (Oder), Müllroser Chaussee 7, D-15236 Frankfurt (Oder) Markendorf, Germany.

Received March 15, 1996

Accepted July 23, 1996

Clinical Study not agree with the opinion of Redondo-Lopez et al.[5] who stated that the clinical picture of *Torulopsis* (*C. glabrata*) vulvovaginitis was not different from the classical presentation of candida vulvovaginitis. The rate of recovery from acute vaginal candidosis is 75–80% after 4 weeks.[6] In the case of chronically recurrent vulvovaginal candidosis (at least 4 episodes per year), the rate is only 50% despite antimycotic treatment carried out over several months.[7]

Proceeding from these facts, we carried out the following investigations to see if immunologic deficiencies in women with acute or chronic *C. albicans* or *C. glabrata* vulvovaginitis could be measured peripherally and possibly eliminated. Some results of our investigations have already been published,[8] specifically those concerning local immunity.[9,10]

### SUBJECTS AND METHODS

### Identification of Yeast Species

Vaginal secretions were collected with a speculum during the vaginal examination and placed on Petri plates containing Sabouraud glucose 2% agar. The specimen was incubated for at least 2 days at 28°C. A positive culture was further differentiated by means of rice-extract agar to identify *C. albicans* by chlamydospores and other species by additional API 20 C auxanogram (Bio Mérieux, Marcy-l'Etoile, France).

### Quantification of T Lymphocytes

A sample of venous blood was taken with a heparinized syringe from each patient prior to any treatment. A sample of heparinized blood was also taken during and after treatment.

The peripheral blood was diluted with an equal volume of RPMI 1640 and layered over Fiquol-Hypaque. The preparation was centrifuged at 400*g* for 30 min to allow density separation of the leukocytes. The layer containing the lymphocytes was harvested and washed. The cells were resuspended at a cell density of approximately 10 cells/ml. An aliquot of 50 μl of this suspension was added to each of 7 tubes and incubated with the monoclonal antibodies against CD4, CD8, CD16, CD19, HLA-DR, CD11b, and an isotopic control antigen (Becton Dickinson, Mountain View, CA) for 30 min. Thereafter, the cells were washed and a sheep anti-mouse FAB fragment, conjugated with fluoroisothiocyanate, was added. After incubation with the fluorescent antibody for 30 min, the cells were washed again. The pellet was fixed with 0.02% formaldehyde in phosphate buffered saline (PBS) and the sample was then run through the flow cytometer (Ortho, Raritan, NJ).

### Proliferation Tests In Vitro

The lymphocyte proliferation assay was performed on 96-well microplates (Greiner, Germany). For stimulation of the 75–100 $\times$ $10^3$ cells, candida antigen (Pasteur) and the mitogens phytohemagglutinin (PHA) concanavalin (Con) A, and pokeweed as well as tuberculin and tetanus antigen were used. All tests were done in several dilutions of the antigens and quadruplets. Sixteen hours before harvesting, 0.5 μCi of $^3$H-thymidine (Amersham, Arlington Heights, IL) was added. On the fifth day, the cells were collected on filters (Titertech cell harvester) and the radioactivity was measured in an LS 7000 Beckmann scintillation counter. An index for the results was used from the mean values (quadruplets) in counts per minute (cpm) of the experiment, e.g., candida 1:40 and thymopentin added, compared with the mean values of 12 not-stimulated probes without antigen or mitogen.

Thymopentin was added in concentrations of 10,000 to 0.01 ng/ml. As an example of one index, the result for candida 1:40 was 24,000 cpm, control 800 cpm, index 30.

### Clinical Follow-Up During and After Thymopentin (Timunox 100, Cilag GmbH, Sulzbach, Germany) Therapy

The data of 17 of the 18 otherwise healthy women presenting with chronically recurrent vaginal candidosis were subdivided in one group of data obtained 30 days before thymopentin therapy and another group obtained 80 days during and after thymopentin therapy. Thymopentin was injected subcutaneously in dosages of 100 mg in the upper arm twice a week for 10–15 injections. After observing that 15 injections did not raise the T lymphocytes more than 10 and considering the high costs of this therapy, we injected only 10 times. Simultaneously, each patient was treated with one 500-mg clotrimazole vaginal tablet.

The average values of T lymphocytes and subpopulations were calculated as mean values between a first value at the beginning of therapy and a second value at the end of the course of injections.

The relapse rate of vaginal candidosis was noted

TABLE 1. Average values of peripheral immunocells in women with and without vaginal mycosis

| | Immunocells (× 10⁶/ml) | | | |
|---|---|---|---|---|
| | | *C. albicans* vulvovaginitis | | |
| | Healthy women (N = 12) | Acute (N = 20) | Chronic (N = 42) | *C. glabrata* vaginitis (N = 14) |
| Lymphocytes (total) | 2.05 +/− 0.69 | 1.94 | 1.88 | 2.21 |
| T lymphocytes | 1.41 +/− 0.49 | 1.18 | 1.25 | 1.43 |
| Precursors | 0.22 +/− 0.14 | 0.21 | 0.25 | 0.29 |
| T suppressor cytotoxic cells | 0.57 +/− 0.27 | 0.50 | 0.48 | 0.51 |
| T helper cells | 0.83 +/− 0.23 | 0.76 | 0.77 | 0.89 |
| B cells | 0.19 +/− 0.07 | 0.16 | 0.19 | 0.21 |
| HLA-DR-carrying cells | 0.41 +/− 0.16 | 0.42 | 0.42 | 0.46 |
| NK cells | 0.61 +/− 0.32 | 0.35* | 0.41** | 0.44*** |

*P = 0.01.
**P = 0.06.
***P = 0.14.

over a period of at least 6 months determined by complaints and clinical signs as well as wet-mount preparations and mycologic cultures of the vaginal secretions about every 4 weeks.

A cure or reduction of the relapse rate was defined as being without symptoms of vulvovaginal candidosis for at least 5 months in a period of 6 months after the end of therapy.

### RESULTS

### Vaginal *C. glabrata* Vaginitis

The number of T-lymphocyte populations did not differ significantly from the values found in healthy women. In fact, the number of natural killer (NK) cells was conspicuously lower.

### Vaginal *C. albicans* Vulvovaginitis

Only the NK cell count in the blood was significantly lower in acute cases compared with healthy women, while all other peripheral immunocells revealed no significant changes.

### *Chronically Recurrent* C. albicans *Vulvovaginitis*

With these women, the peripheral NK cell count was less significantly lower in comparison with healthy women. These results are summarized in Table 1.

### Proliferation Tests In Vitro

In the 16 women with chronically recurrent *C. albicans* vulvovaginitis, only 5 cases revealed a significant and 3 cases a weak reaction to candida antigen which, in initial tests, carried out with different commercial and some homologue candida antigens had been particularly stimulating.[11] Therefore, further comparative proliferation tests were carried out.

Thus, 3 women with acute and 8 women with chronically recurrent *C. albicans* vaginitis as well as 1 woman with *C. glabrata* vaginitis could be compared with 6 healthy women. The lymphocytes showed a good reaction to mitogens in all women. However, the reaction was better in the healthy women. The different antigen stimulants showed lower single and average values of indices. To see if patients more frequently showed a weak reaction to candida antigen or additional thymopentin stimulation than healthy women, we defined a weak proliferate T-lymphocyte reaction as a lower limit of the corresponding index (3 or 2). However, we could not make a conclusion from the present results (Table 2).

### Results In Vivo During and After Thymopentin Therapy in Women Presenting With Chronically Recurrent *C. albicans* Vulvovaginitis

On average, the treatment with thymopentin resulted only in a low mean increase of T lymphocytes and B cells, while the number of T-helper cells, T-suppressor cytotoxic cells, and NK cells decreased, which seem contradictory. An individual consideration of the values of each patient by means of an index is more revealing. This index was defined by noting down the number and increase of the respective immunocells before and after treatment of each patient. It revealed a correlation between

TABLE 2. Proliferation tests in vitro: Distribution of weak proliferations (index values) within various patient groups

| | Mitogens (index ≦3) | Tetanus (index ≦2) | Tuberculin (index ≦2) | Candida (index ≦2) 1:5 | Candida (index ≦2) 1:40 | Candida + thymopentin (index ≦2) 1:5 | Candida + thymopentin (index ≦2) 1:40 |
|---|---|---|---|---|---|---|---|
| Chronically recurrent *C. albicans* vaginitis (N = 8) | 0/8 | 2/8 | 5/8 | 5/8 | 4/8 | 5/7 | 5/7 |
| Acute *C. albicans* vulvovaginitis (N = 3) | 2/3 | 0/3 | 2/3 | 3/3 | 0/3 | 3/3 | 2/3 |
| *C. glabrata* vaginitis (N = 1) | 0/1 | 0/1 | 1/1 | 1/1 | 0/1 | 0/1 | 0/1 |
| Healthy women (N = 6) | 0/6 | 1/6 | 3/6 | 4/6 | 3/6 | 4/6 | 3/6 |

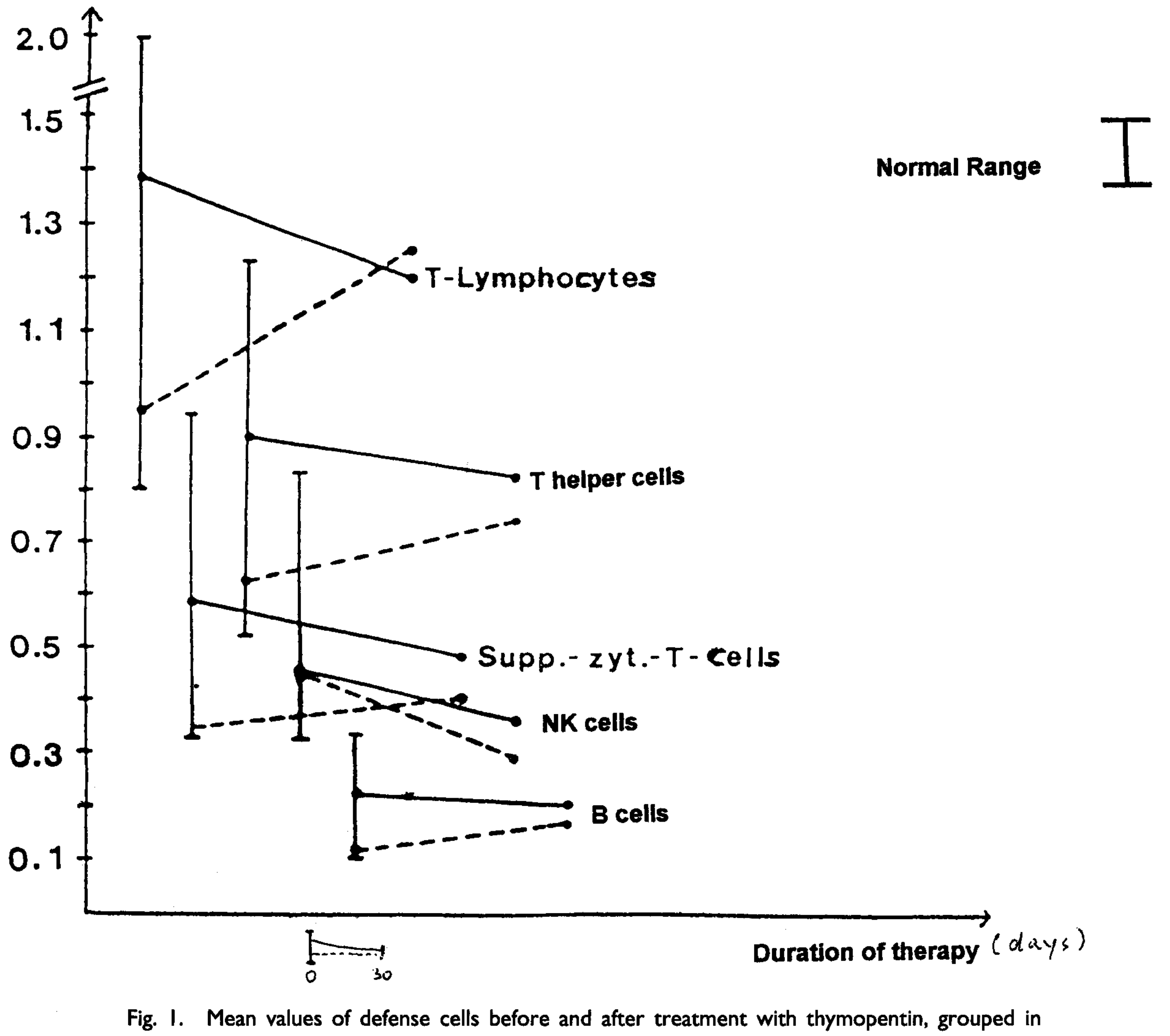

Fig. 1. Mean values of defense cells before and after treatment with thymopentin, grouped in patients with (- - -) and without (——) successful therapy. Supp.-zyt. = suppressor-cytotoxic.

a decreasing index and unsuccessful therapy and vice versa. This trend was also suggested by the average values of all women with and without successful therapy. The changes often occurred within the (lower) normal limits. A graphic representation of the development, however, also revealed differ-

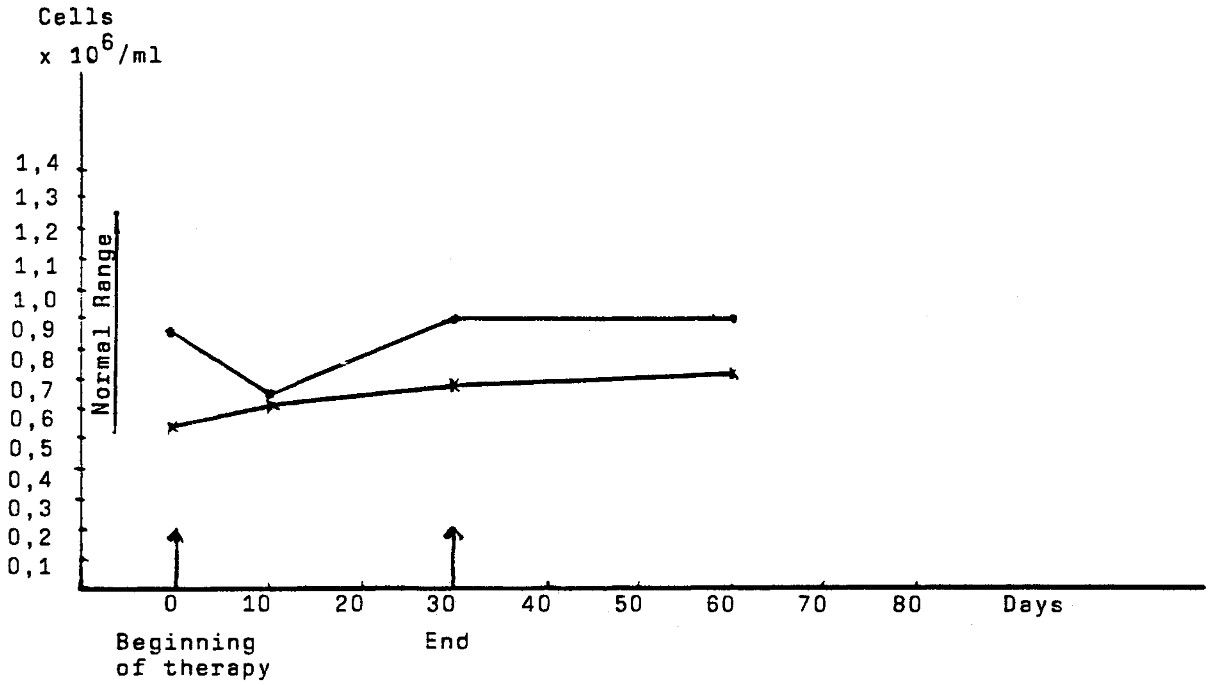

Fig. 2. Clinical course: average values of the helper cells of (•) 10 non-cured patients and (×) 8 cured patients before and after immunostimulation with thymopentin.

ences between patients with and without cure (Fig. 1). In women who were cured, the average values of T-helper cells were low, primarily within the lower normal range, and slightly increased subsequent to therapy. In the case of unsuccessful therapy, the normal average values of T-helper cells, despite thymopentin injection, primarily decreased within the first 1 or 2 weeks, returning to the initial values after about 4 weeks (Fig. 2).

### Clinical Results After Thymopentin Therapy

In all, 18 women were treated with adjuvant thymopentin. Sixteen of these patients suffered from chronically recurrent vaginal candidosis, of whom 15 were caused by *C. albicans* and 1 was caused by *C. krusei.* One patient could not be evaluated because of no data. The 18th patient had allegedly suffered from vaginal infection caused by *C. glabrata* for 9 years; however, *C. albicans* was also demonstrable on one occasion.

Before therapy, 16 women suffering from typical vaginal candidosis for an average of 3.9 years were evaluable. In 9 cases, the number of T lymphocytes before therapy was within or under the lower normal range. In 6 of these patients, thymopentin treatment resulted in a cessation of relapses, which was defined as being without symptoms of vulvovaginal candidosis for at least 5 of 6 months. Of the remaining 7 women who, before therapy, showed normal numbers of T cells, only 1 patient was treated successfully, while the other 6 suffered from relapses as before. The patient with *C. glabrata* vaginitis had normal values of T lymphocytes before and after the treatment; however, her therapy was not successful.

### DISCUSSION

### Cellular Defense in Vaginal Mycoses

On average, the women with acute and chronically recurrent vaginal candidosis—frequently with the normal range of values—had fewer T lymphocytes, fewer T-helper cells, fewer NK cells, and fewer B cells than healthy women. Numerous references point to the significance of cellular defense in the case of candida infection.[12–19]

The association of slightly lowered T-helper cells with vaginal candidosis was proven in a group of immune-suppressed patients. NK cells and a variety of cytokines both seem to play their parts, apparently in the defense against *C. albicans* as well.[20] Phagocytosis may vary, however, depending on the yeast species,[21] strain properties,[22] or protease activity.[23]

Vaginal candidosis seems to lead to frequent blocking of defense cell activity, a phenomenon that was also shown in other investigations.[17,24–26] Humoral system defects result in an absence of opsonization, an absence of complement formation, and an absence of T cells, while cellular-system defects adversely affect cytotoxic T cells, lymphokine production, and phagocytosis or the presentation of antigens. Thus, an answer regarding the relationship of cause and effect in candida infections will remain a mystery for some time: "In some instances, however, the cause and effect relationship is not clear, i.e., did the infection with candida initiate the immunosuppression, or did the underlying condition result in immunosuppression allowing for candida to initiate disease?"[27]

### Immunostimulation by Means of Thymopentin

Although the slgA in the cervicovaginal secretions could essentially be increased by thymopentin,[10] the therapeutic results could not be improved. This observation emphasizes the importance of the cellular component with regard to resistance in vaginal mycoses. The proliferation tests in vitro revealed different reactions to candida antigens, depending on the individual patient.

Mathur et al.[28] observed, in women with chronic vaginal candidosis, normal reactions to PHA, which is more stimulating to T-helper cells, but only weak reactions to Con A, which is more stimulating to the T-suppressor cytotoxic cells. There was a significant correlation between the depression and high anti-candida antibody titers in the serum. Witkin et al.,[29] as we did, found no differences between women with and without vaginal candidosis concerning normal T-cell proliferation to mitogens, but a weak response to candida antigen. The sera of these patients suppressed the proliferation of control T lymphocytes against candida antigen.

Two reaction types were apparent in our patient group. One group of women with their numbers of lymphocytes lying primarily within the middle or upper normal range who, after candida and thymopentin stimulation in vitro, revealed no increased proliferations, although the unspecific mitogen stimulation was normal and the lymphocytes after thymopentin application in vivo were rather decreased. These women, in most cases, were not cured from chronically relapsing vaginal candidosis. Another group consisted of women presenting with primarily low numbers of T lymphocytes, which could be increased by thymopentin and well stimulated in vitro by candida antigen and thymopentin. These women were cured.

### REFERENCES

1. Ghannoum MA, Abu-Elteen KH: Pathogenicity determinants of *Candida*. Mycoses 33:265–282, 1990.
2. Krempl-Lamprecht L: Zur mikrobiologischen Charakteristik von *Candida albicans*. Int Exp Clin Chemother Suppl 1:16–20, 1990.
3. Mendling W: Die Vaginalcandidose. Dtsch Ges Gynäkol Geburtsh Mitteilungen 4:209–210, 1991.
4. Mendling W: Die Torulopsidose in der Frauenheilkunde. Geburtshilfe Frauenheilkd 44:583–586, 1984.
5. Redondo-Lopez V, Lynch M, Schmidt C, Cook R, Sobel JD: *Torulopsis glabrata* vaginitis: Clinical aspects and susceptibility to antifungal agents. Obstet Gynecol 76:651–655, 1990.
6. Cohen L: Is more than one application of an antifungal necessary in the treatment of acute vaginal candidosis? Am J Obstet Gynecol 152:961–964, 1985.
7. Sobel JD: Management of recurrent vulvovaginal candidiasis with intermittent ketoconazole prophylaxis. Obstet Gynecol 65:435–440, 1985.
8. Mendling W, Koldovsky U: Immunological findings in patients with chronically recurrent vaginal candidosis and new therapeutic approaches. Mycoses 32:386–390, 1989.
9. Mendling W, Metzger P: Bestimmung des sekretorischen Immunglobulin A im Zervikovaginalsekret gesunder Frauen mit einer ELISA-Methode. Geburtschilfe Frauenheilkd 54:417–420, 1994.
10. Mendling W, Koldovsky U: Further immunological investigations in vaginal mycoses. Mycoses 39:177–183, 1996.
11. Koldovsky U, Mendling W: Die proliferative Antwort von Lymphozyten Candidaerkrankter auf Stimulation mit verschiedenen Candidaantigenen. IV. Intern Workshop Infekt Gyn Geburtsh, München, 1989.
12. Valdimarsson H, Higgs JM, Wells RS, Yamamura M, Hobbs JR, Holt L: Immune abnormalities associated with chronic mucocutaneus candidiasis. Cell Immun 6:348–361, 1973.
13. Fegeler K, Macher E, Nolting S: Tierexperimentelle Untersuchungen zur Immunität bei Infekten mit *Candida albicans*. Mykosen 21:127–137, 177–189, 201–222, 1978.
14. Meinhof W: Candida—Mykosen und celluläre Immundefekte. Z Haut Geschlechtskr 141:1–10, 1979.
15. Coegniet E, Kühnast R: Rezidivierende Candidiasis. Adjuvante Chemotherapie mit verschiedenen Echinacin-Darreichungsformen. Therapiewoche 36:3352–3358, 1986.
16. Török J, Farkas B: Die Bedeutung der Phagozyten und des T-Lymphozyten-Systems bei der Abwehr von *Candida albicans*—Infektionen (Übersicht). Mykosen 29: 486–490, 1986.

17. Dwajari D, Simon M, Burkert B: Macrophage functions in patients suffering from chronic mucocutaneous candidiasis. Arch Dermatol Res 275:412–416, 1983.
18. Witkin SS, Hirsch J, Ledger WJ: A macrophage defect in women with recurrent *Candida* vaginitis and its reversal in vitro by prostaglandin inhibitors. Am J Obstet Gynecol 155:790–795, 1986.
19. Sinha BK, Monga DP, Prasad S: Studies on the role of macrophages in experimental candidosis in mice. Mykosen 30:105–112, 1987.
20. Greenfield RA: Host defense system interactions with *Candida.* J Med Veter Mycol 30:89–104, 1992.
21. Vecchiarelli A, Bistoni F, Cenci E, Perito S, Cassone A: In vitro killing of *Candida* species by murine immunoeffectors and its relationship to the experimental pathogenicity. Sabouraudia (J Med Veter Mycol) 23:377–387, 1985.
22. Hauck H, Simon B, Geschwender B, Djawari D: Beeinflußbarkeit von Phagozytose und intrazellulärer Abtötung von *Candida albicans* durch PMNL durch Stammeigenschaften des Erregers. Z Haut Geschlechtskr 150:660–666, 1985.
23. Walther T, Rytter M, Schönborn C, Haustein UF: Differences in the intracellular killing of proteinase-positive and proteinase-negative *Candida albicans* strains by granulocytes. Mykosen 29:159–161, 1986.
24. Piccolella E, Lombardi G, Morelli R: Human lymphocyte activating properties of a purified polysaccharide from *Candida albicans:* B- and T-cell cooperation in the mitogenic response. J Immunol 125:2082–2088, 1980.
25. Melbye M, Schonheyder H, Kestens L, Stenderup A, Gigase PL, Ebbesen P, Biggar RJ: Carrige of oral *Candida albicans* associated with high number of circulating suppressor-T-lymphocytes. J Infect Dis 152:1356–1357, 1985.
26. Danley DJ, Polakoff J: Rapid killing of monocytes in vitro by *Candida albicans* yeast cells. Infect Immun 51:307–313, 1986.
27. Domer JE, Garner RE: Immunomodulation in response to *Candida.* In Kustak E (ed): Immunology of Fungal Diseases. New York: Marcel Dekker, Inc., 1989.
28. Mathur S, Mathur RS, Dowda H, Williamson O, Faulk WP, Fudenberg HH: Sex steroid hormones and antibodies to *Candida albicans.* Clin Exp Immunol 33:79–87, 1978.
29. Witkin SS, Yu R, Ledger WJ: Inhibition of *Candida* induced lymphocyte proliferation by lymphocytes and sera from women with recurrent vaginitis. Am J Obstet Gynecol 147:809–811, 1983.
